# Supplementary material for: Roles of AGD2a in Plant Development and Microbial Interactions of Lotus japonicus
Source: Int J Mol Sci. 2022 Jun 20;23(12):6863. doi: 10.3390/ijms23126863 (PMC9224730; doi:10.3390/ijms23126863)
Supplement: Supplementary file 1 [file ijms-23-06863-s001.zip › Figure S-Roles of AGD2a in plant-ijms-1747451.pdf]

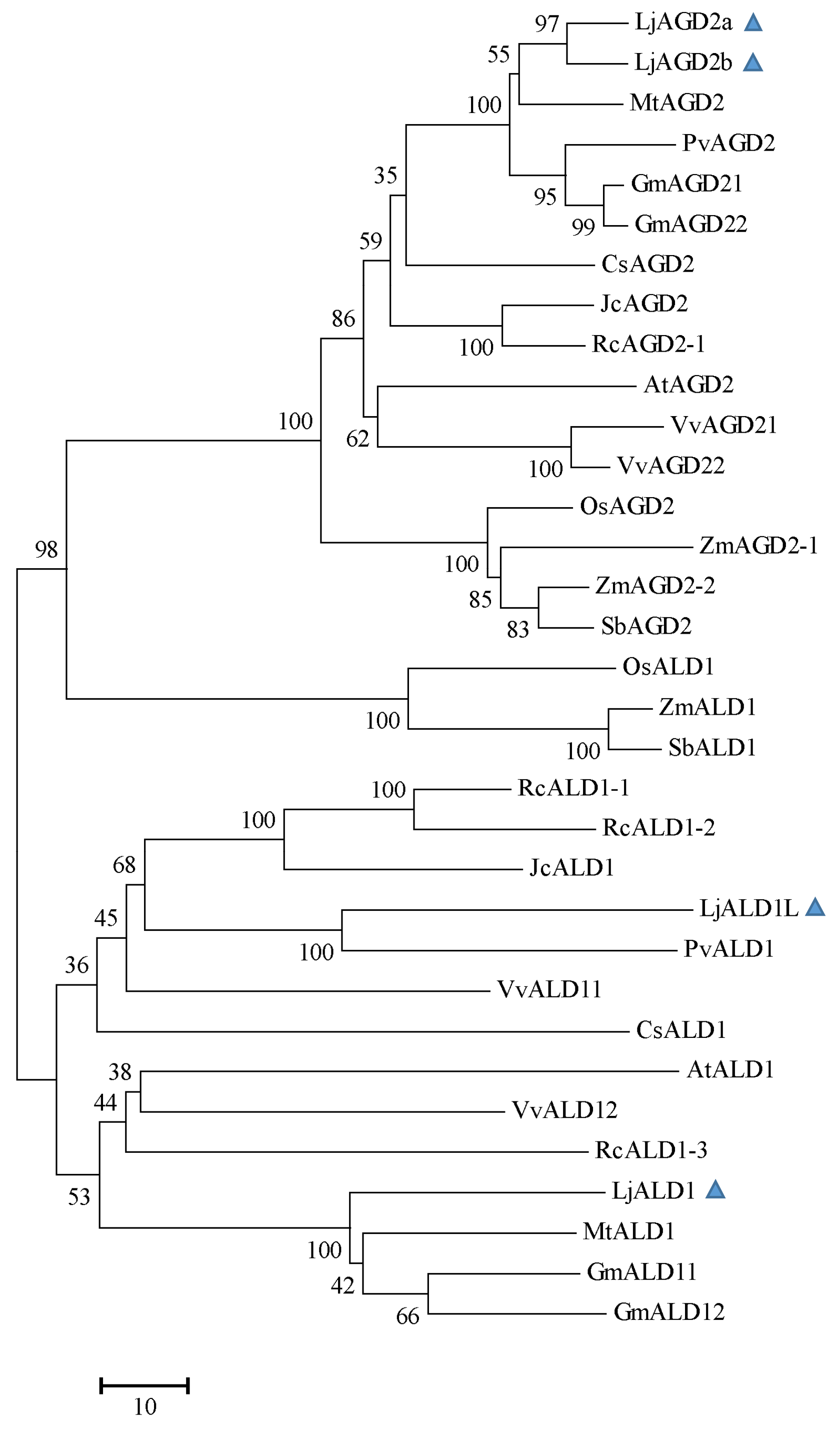

Figure S1. Phylogenetic tree of *AGD2* family genes. Sequences were obtained using sequences retrieved from Phytozome (http://www.phytozome.net/), NCBI (http://www.ncbi.nlm.nih. gov/) and Kazusa (http://www.kazusa.or.jp/lotus/) databases with LjALD1 as query. Protein sequences were aligned by ClustalX v1.83 software with default settings. Unrooted tree calculated using the neighbor‐joining method in MEGA version 6 with 1000 bootstrap replicates (Tian et al. 2017; Tamura et al. 2013). Accession numbers of the AGD2 protein included in the analysis: *Lotus japonicus* (LjALD1, Lj6g3v2218710.1; LjALD1L, Lj1g3v0652040.1; LjAGD2a, Lj6g3v1078410.1; LjAGD2b, Lj4g3v0412700.1), *Medicago truncatula* (MtALD1, Medtr2g008430.1; MtAGD2, Medtr4g092620.1), *Glycine max* (GmALD1-1, Glyma.08G180600.1; GmALD1-2, Glyma.15G051800.1; GmAGD2‐1, Glyma.07G185700.1; GmAGD2‐2,

Glyma.08G063500.1), *Arabidopsis thaliana* (AtALD1, AT2G13810; AtAGD2, AT4G33680), *Vitis vinifera* (VvALD1 - 1, GSVIVT01008968001; VvALD1 - 2, GSVIVT01029761001; VvAGD2 - 1, GSVIVT01036981001; VvAGD2 - 2, GSVIVT01036983001), *Phaseolus vulgaris* (PvALD1, Phvul.008G169600.1; PvAGD2, Phvul.002G217100.1), *Oryza sativa* (OsALD1, LOC_Os03g09910.1; OsAGD2, LOC_Os03g18810.1), *Zea mays* (ZmALD1, GRMZM2G119150_T01; ZmAGD2 - 1, GRMZM2G415117_T01; ZmAGD2 - 2, GRMZM2G010328_T01), *Cucumis sativus* (CsALD1, XP_011649917; CsAGD2, XP_004147517), *Jatropha curcas* (JcALD1, KDP32718; JcAGD2, KDP35908), *Sorghum bicolor* (SbALD1, Sobic.001G471100.1; SbAGD2, Sobic.001G401700.1), *Ricinus communis* (RcALD1-1, 29908.m005969; RcALD1-2, 29923.m000815; RcALD1-3, 29195.m000052; RcAGD2, 30099.m001639). The scale bar corresponds to 10 estimated amino acid substitutions per site. Blue triangles indicate AGD2 homologs in *L. japonicus*.

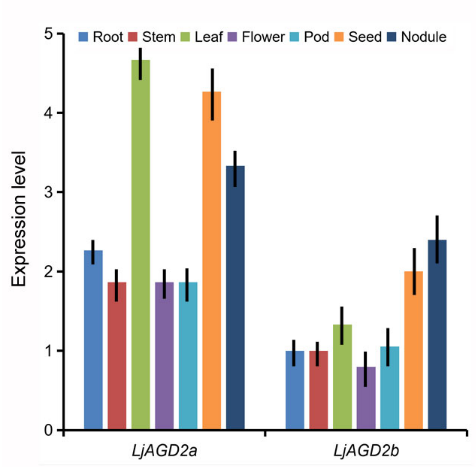

Figure S2. Expression patterns of *LjAGD2a* and *LjAGD2b* in *L. japonicus*. Error bars represent the SD of means obtained from analyses of three biological replicates.

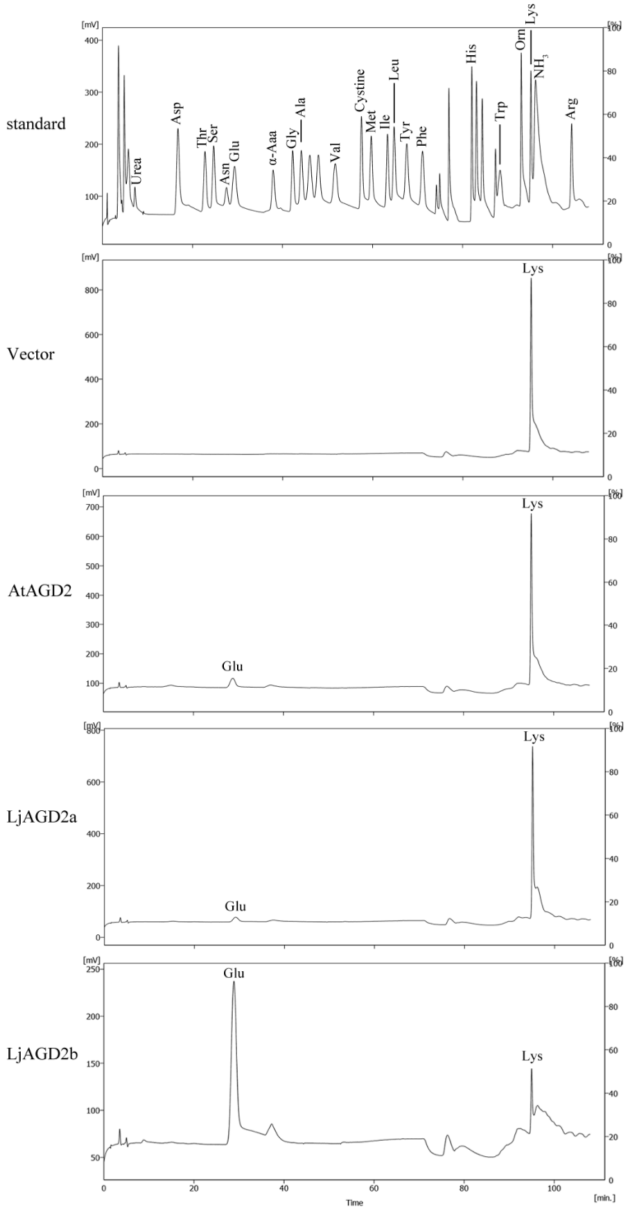

Figure S3. Liquid chromatograms of amino acids demonstrating the aminotransferase function of LjAGD2a and LjAGD2b *in vitro*, generated in reaction mixtures consisting of 50 mM Tris HCl (pH 8.0), 50 mM Lys, 50 mM 2-OG, 1 μM PLP and 100 mM MgCl2. After reaction at 37 °C for 6 h, products were detected by an amino acid analyzer. In each case the left ordinate indicates the voltage (mV), the right ordinate indicates amino acid contents (%), and the abscissa indicates peak elution times (min). From the top, chromatograms of: mixed amino acid standard (with peaks of common amino acids marked) and reaction mixtures obtained with empty vector, AtAGD2, LjAGD2a and LjAGD2b. When AtAGD2, LjAGD2a and LjAGD2b were present, Glu was formed in the products.

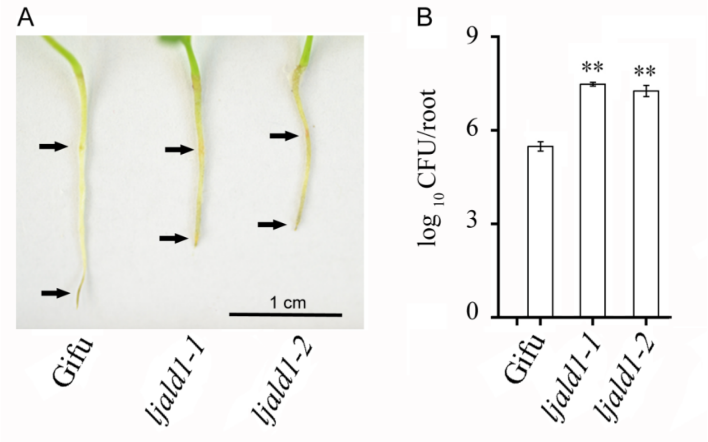

Figure S4. *ljald1* mutants were more susceptible than wildtype plants to *Ralstonia solanacearum*. (A) Photograph of Gifu and *ljald1* mutants 5 days after infection of roots by *R. solanacearum*. The bottom parts of roots of each line became brown, but as indicated by the arrows roots of *ljald1* mutants became significantly deeper brown than Gifu roots. (B) Growth curves of *R. solanacearum* strains on the roots. The indicated genotypes were inoculated with *R. solanacearum* with a bacterial suspension of OD_{600} = 0.0002 and growth of bacteria was assayed 5 days after infection. n = 12 plants in each line were used for observation and error bars represent the SD of 3 replicates.. (** indicates P < 0.01 according to the Duncan test).

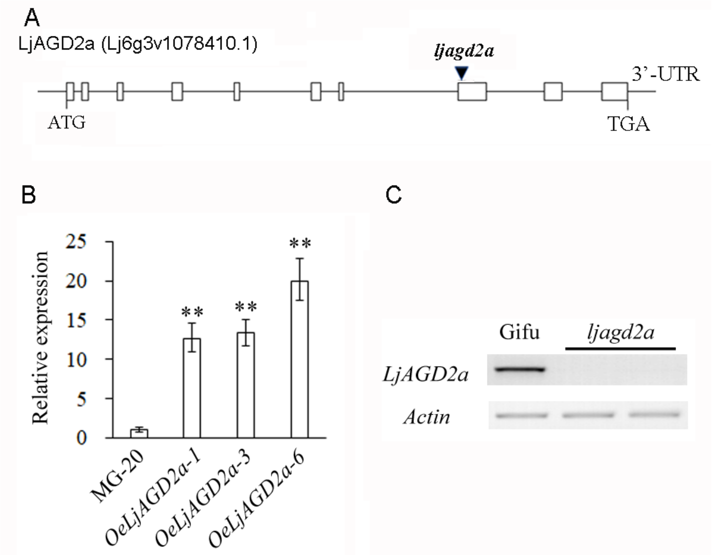

Figure S5. *LjAGD2a* gene expression patterns. (A) Gene structure of *LjAGD2a*. The box, black line and black triangle indicate the exon, 5 '- UTR, 3' - UTR or intron and insertion position of the *LORE1* mutant. Positions of the ATG start codon and TGA stop codon are marked. (B) Relative expression levels of *LjAGD2* in leaves of MG-20 and *OeLjAGD2a* plants. Error bars represent the SD of 3 replicates.. ** indicates a significant difference (P < 0.01) compared with wild type MG-20 plants according to Student's t test. (C) *LjAGD2a* gene expression levels in leaves of wildtype (Gifu) and *ljagd2a* mutant plants. There were 26 cycles of *Actin* primer amplification and 32 cycles of *LjAGD2a*-specific primer amplification.
